# Genomic Insights into the Genetic Diversity, Selection Signals, and Agronomic Traits of Rice (*Oryza sativa* L.) Germplasm in Zhejiang Province

**DOI:** 10.3390/plants15152368

**Published:** 2026-07-31

**Authors:** Yang Lv, Hao Wu, Muhammad Asad Ullah Asad, Guoyong Liu, Mingming Wu, Jing Ye, Rongrong Zhai, Shenghai Ye, Xiaoming Zhang, Faming Yu

**Affiliations:** 1 Institute of Crop and Nuclear Technology Utilization, Zhejiang Academy of Agricultural Sciences, Hangzhou 310021, China; lvyang0725@163.com (Y.L.);; 2 Guangxi Key Laboratory of Rice Genetics and Breeding, Rice Research Institute, Guangxi Academy of Agricultural Sciences, Nanning 530007, China; 3 Zhejiang Wunong Seed Industry Co., Ltd., Lishui 323406, China

**Keywords:** rice, genome, genetic diversity, GWAS, haplotype analysis

## Abstract

Elucidating the evolutionary trajectories and genetic basis of critical agronomic traits in regional rice germplasm is paramount for discovering elite allelic variations for crop improvement. Here, we systematically characterized a panel of 109 rice accessions from Zhejiang Province through whole-genome resequencing (~10× coverage) coupled with two years of rigorous field phenotypic evaluations. A total of 4,753,071 high-quality genomic variants, including 4,147,316 SNPs, were identified across the genome. Population structure and evolutionary analyses revealed sharp genetic differentiation at the subspecies level, partitioning the panel into distinct *indica* and *japonica* clusters accompanied by intricate subpopulation stratification and historical gene flow. Through a joint scanning of the fixation index (*F*st) and nucleotide diversity (*Pi*) ratios, three prominent selective sweep regions (*qSS1*, *qSS10*, and *qSS12*) driving subspecific differentiation were captured on chromosomes 1, 10, and 12. Notably, the *qSS12* locus harbors the sucrose transporter gene *OsSUT2*, indicating that carbohydrate transport and energy metabolism served as core genomic targets driving the indica–japonica divergence. Furthermore, genome-wide association studies (GWAS) successfully mapped 9 significant loci modulating heading date, effective tiller number, and grain size. Subsequent gene-based haplotype analyses within these target intervals pinpointed elite allelic variations in core candidate genes, including *OsSPX1* (phosphate homeostasis, 1000-grain weight), Chl9 (chlorophyll synthesis, grain width), and *OsCER1* (wax biosynthesis, panicle length). Collectively, this study deciphers the genomic landscape and subspecies differentiation patterns of Zhejiang rice germplasm, providing pivotal molecular targets and invaluable genomic resources for germplasm conservation and precision molecular breeding.

## 1. Introduction

Rice (*Oryza sativa* L.) stands as the most consequential cereal crop underpinning the dietary caloric requirements of over half of the world’s inhabitants; consequently, targeted genetic enhancement of its agronomic performance and end-use quality represents an imperative for sustaining global nutritional security [1,2]. Situated in the core of the Yangtze River Delta, Zhejiang Province has long been one of the primary rice-producing regions in China, favored by its superior climatic conditions and fertile arable land [3]. In addition to the high-yielding modern cultivars currently under extensive cultivation, this province harbors a vast reservoir of traditional native landraces. Through long-term natural and artificial selection, these landraces have progressively enriched unique genetic variations underlying localized ecological adaptation, biotic/abiotic stress tolerances, and superior agronomic performances, thereby constituting an indispensable component of the rice germplasm system [4,5]. Consequently, a systematic evaluation of the genetic diversity, characterization of the population structure, and dissection of the pivotal genes modulating key agronomic traits in Zhejiang rice germplasm hold profound theoretical and practical value for both germplasm conservation and breeding utilization.

The genetic diversity and population structure of germplasm resources represent the fundamental prerequisites for driving genetic research and cultivar improvement [6,7]. The genetic divergence between *indica* and *japonica* subspecies of rice has been extensively characterized through earlier investigations that harnessed PCR-based simple sequence repeat (SSR) polymorphisms and chip-based single nucleotide polymorphism (SNP) genotyping platforms [8]. More recently, the maturation of high-throughput sequencing technologies has positioned whole-genome resequencing as an indispensable instrument for the high-resolution dissection of intraspecific genomic variation, the inference of population evolutionary dynamics, and the genome-wide localization of agronomically relevant loci [9,10]. Leveraging this methodology, genome-wide association studies (GWAS) have become a highly effective strategy for dissecting the genetic architecture of complex crop traits, providing an abundance of potential genetic targets for marker-assisted selection [11,12,13]. By integrating high-density molecular markers with precise phenotypic evaluations, GWAS can effectively detect significant association loci and candidate genes governing yield, growth period, heading date, plant architecture, and grain quality [14,15]. Nevertheless, most current resequencing studies remain focused on national or global scales of germplasm collections [16], leaving regional germplasms with unique agricultural and ecological significance—such as those from Zhejiang Province—largely uncharacterized from a systematic genomics perspective. Accumulating evidence indicates that local landraces frequently harbor beneficial allelic variations related to stress resistance, grain quality, and adaptability that have been progressively lost in modern cultivars [17]. Therefore, there is an urgent need to deploy whole-genome resequencing to perform high-resolution population genetics and association analyses on Zhejiang local resources, aiming to unlock elite alleles that have not yet been fully exploited by modern breeding programs.

Concurrently, although Zhejiang rice germplasm resources exhibit significant potential in breeding and agricultural practices, substantial gaps remain regarding the in-depth analysis of their genetic diversity and the molecular mechanisms underlying key agronomic traits. To address these limitations, this study investigated a representative panel of 109 rice accessions from Zhejiang Province, encompassing traditional landraces and modern cultivars. High-accuracy SNP data were generated via whole-genome resequencing (at approximately 10× coverage), which were subsequently integrated with two years of rigorous field phenotypic data (including plant height, tiller number, growth period, grains per panicle, seed setting rate, and grain size) to systematically dissect the population structure and genomic variation profiles. On this basis, multidimensional bioinformatic approaches—including GWAS, selective sweep analysis, and gene-based haplotype analysis—were implemented to identify critical genomic regions associated with major agronomic traits and screen for functionally relevant candidate genes. This work establishes a scientific foundation for subsequent gene functional validation and its applications in rice genetic improvement. Ultimately, this study not only enriches the genomic information of regional rice germplasm but also provides essential theoretical support for formulating more precise and efficient molecular design breeding strategies.

## 2. Results

### 2.1. Variant Detection and Characterization

**Figure 1:**
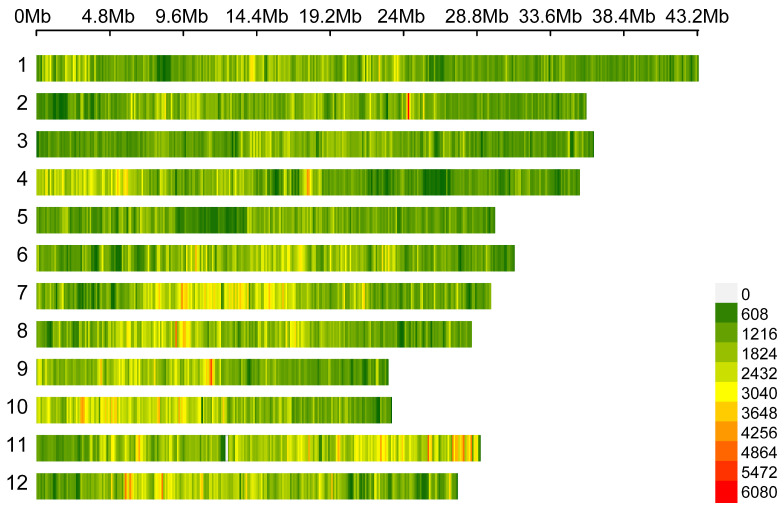
Genome-wide distribution of SNP and Indel densities across 12 rice chromosomes. SNP and Indel counts per 0.1 Mb non-overlapping sliding window.

The resequencing of 109 geographically diverse Zhejiang rice accessions produced a comprehensive catalog of 4,753,071 high-quality genomic variants after the application of stringent filtering thresholds (MAF > 0.05; missing rate < 20%). This resource encompassed 4,147,316 SNPs and 605,755 Indels distributed evenly across the rice genome’s 12 chromosomes, with chromosome-level densities spanning 0.92–15.93 variants/kb (Figure 1). Notably, chromosome 1 harbored the highest abundance of variants with 518,917 variants (average density of 12.00 variants/kb), whereas chromosome 9 contained the fewest with 298,478 variants (average density of 12.98 variants/kb) (Supplementary Table S1). Genomic functional annotation of the high-quality variant set revealed that intergenic regions and flanking upstream/downstream regions constituted the vast majority (84.78%). Concurrently, synonymous and missense variants accounted for 2.48% and 3.30% of the dataset, respectively, whereas frameshift mutations (0.23%) and stop-lost variants (0.02%) were less abundant (Supplementary Table S2).

### 2.2. Phenotypic Variation Analysis

The 10 major agronomic traits evaluated exhibited rich genetic variation within the 109 Zhejiang rice accessions (Table 1). The coefficient of variation (CV) for individual traits ranged from 9.39% for the total growth period to 34.64% for the effective tiller number, reflecting diverse degrees of phenotypic dispersion across the population. Specifically, high CV values were observed for the effective tiller number (CV = 34.64%) and the number of grains per panicle (CV = 26.76%), highlighting the significant potential of these two core yield components for targeted artificial selection in Zhejiang regional germplasm. Conversely, the growth period (CV = 9.39%) and seed setting rate (CV = 9.43%) displayed relatively narrow variation, suggesting highly consistent performance within the population.

The frequency distribution histograms of all traits approximately followed a normal distribution (Figure S1), with no obvious bimodal distribution, satisfying the basic assumptions of genome-wide association study (GWAS) for phenotypic data. Furthermore, the Shannon–Wiener genetic diversity index ranged from 1.827 for panicle length to 2.012 for the effective tiller number. Notably, the diversity indices for the effective tiller number, seed setting rate, and grain length surpassed 2.00, demonstrating high genetic richness within this germplasm collection. Collectively, the robust phenotypic variations and broad genetic base of the 109 accessions establish a highly reliable foundation for the subsequent mapping of agronomic QTLs and functional candidate genes.

### 2.3. Population Structure and Phylogenetic Relationships

**Figure 2:**
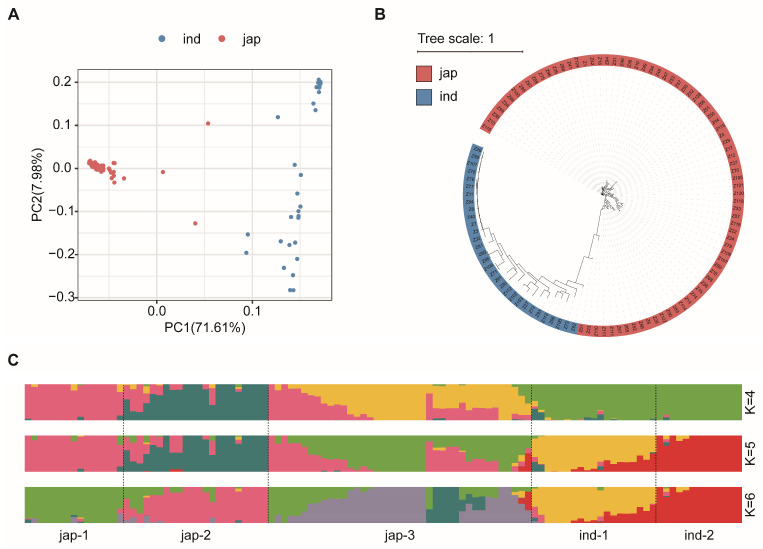
Population structure and phylogenetic relationships of 109 Zhejiang rice accessions. PCA (**A**), phylogenetic tree (**B**), and ADMIXTURE ancestry plots at K = 4–6 (**C**) showing *indica*–*japonica* divergence and subpopulation stratification.

The 109 Zhejiang rice accessions resolved into two distinct genetic clusters corresponding to the indica and japonica subspecies, as evidenced by concordant results from PCA, phylogenetic reconstruction, and ADMIXTURE-based structure analysis (Figure 2). PC1 and PC2 accounted for 71.61% and 7.98% of the genomic variance, respectively, achieving complete spatial separation of the two major germplasm groups and highlighting a robust subspecific differentiation background. The neighbor-joining (NJ) phylogenetic tree consistently validated this dimorphic architecture (Figure 2A,B). Population structure inference modeled via ADMIXTURE indicated that at ancestral cluster values of K = 4 to 6, the indica cluster could be stratified into two distinct subpopulations, while the japonica cluster could be sub-divided into two to three subpopulations (Figure 2C). Although the ancestral composition of most accessions remained relatively pure, several individuals exhibited admixed ancestries, suggesting historical inter-subspecific or inter-subpopulation gene flow. Taken together, these results confirm sharp genetic divergence between the indica and japonica subspecies of Zhejiang rice, with a more intricate subpopulation stratification nested within the japonica germplasm pool.

### 2.4. Genetic Diversity and Selective Sweep Analysis

**Figure 3:**
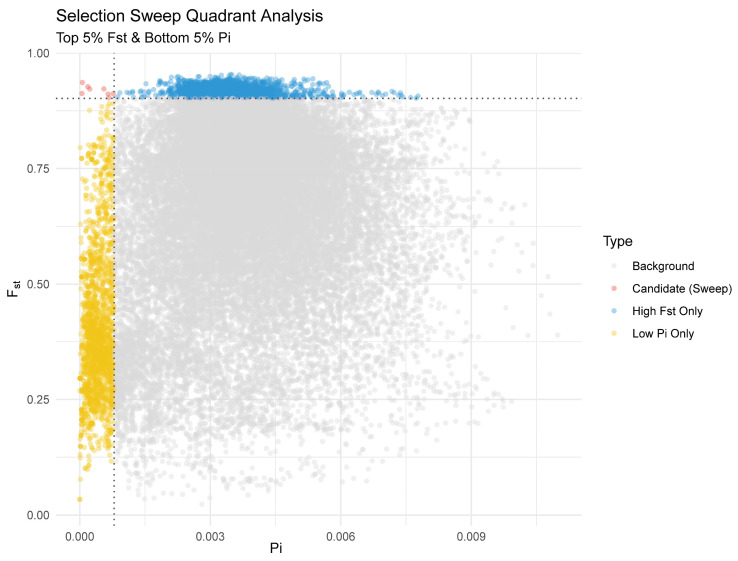
Genome-wide distribution of selective sweep regions identified by *F*st and *Pi* ratios. Manhattan plots of genome-wide *F*st (top) and *Pi* (bottom). Dashed lines: top 5% *F*st (red) and bottom 5% *Pi* (blue).

**Figure 4:**
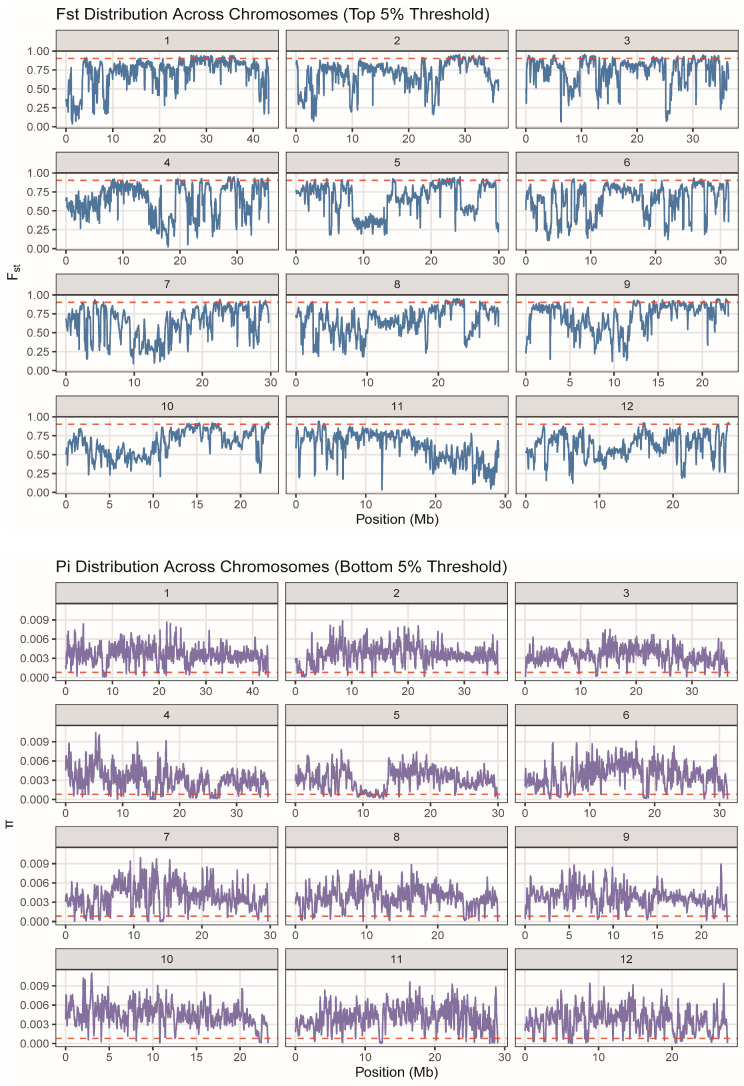
Genome-wide distribution of pairwise *F*st and *Pi* between *indica* and *japonica* subpopulations. Sliding-window *F*st values between indica and japonica. High peaks indicate strong differentiation. Dashed line: top 5% and bottom 5% threshold.

To By jointly scanning the genome-wide fixation index (*F*st) and nucleotide diversity (*Pi*), and using the top 5% of *F*st values combined with the bottom 5% of *Pi* values as the significance thresholds (Figure 3 and Figure 4 and Figure S2), three selective sweep regions were identified on chromosomes 1, 10, and 12, designated as *qSS1*, *qSS10*, and *qSS12*, respectively (Table 2).

*qSS12* consisted of four consecutive windows (Chr12: 27.50–27.63 Mb), with weighted *F*st values ranging from 0.912 to 0.922 and *Pi* values as low as 5.0 × 10^−5^–7.7 × 10^−4^. This interval contained the sucrose transporter gene *OsSUT2* (Supplementary Table S3), which plays a key role in source–sink allocation of photosynthetic products [18,19]. Previous studies have shown that loss-of-function mutants of this gene exhibit dwarfism, reduced tiller number, and decreased 1000-grain weight, suggesting that this locus may have experienced strong artificial selection during high-yield breeding of Zhejiang rice. Mechanistically, *OsSUT2* mediates vacuolar sucrose efflux to the cytosol; its disruption triggers photosynthetic feedback inhibition in source leaves and reduces carbon supply to roots, accompanied by altered hormone balance [19]. The *qSS1* interval was enriched with genes involved in photosynthesis and chloroplast function (e.g., photosystem subunits, ATP synthase). The *qSS10* interval comprised three consecutive windows (Chr10: 23.18–23.30 Mb) and contained the plastid Clp protease gene *CLPP4* (Supplementary Table S3) [20].

**Figure 5:**
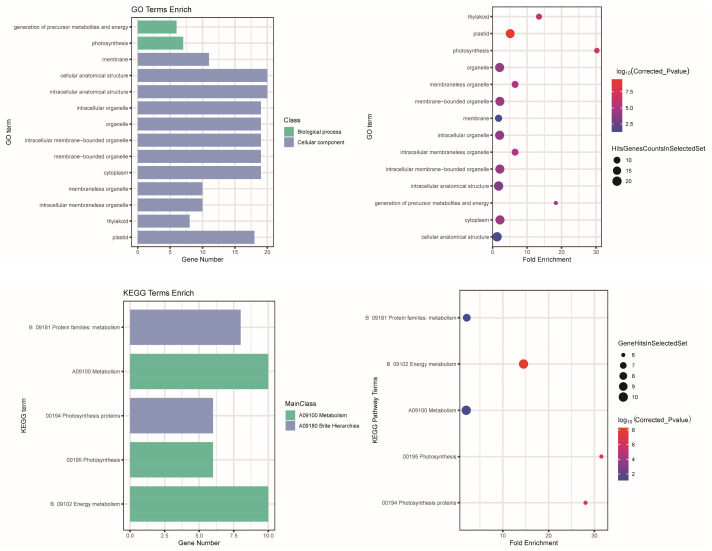
GO and KEGG enrichment analyses of genes located within the selective sweep regions. dot size = gene count.

GO and KEGG enrichment profiling was subsequently employed to interrogate the functional categories of genes captured within the selective sweeps (Figure 5). These analyses disclosed marked enrichment for functional categories implicated in plastid development, thylakoid membrane organization, photosynthesis, and energy metabolism (*p* < 0.05). Collectively, these findings indicate that photosynthetic efficiency, energy metabolism, and sugar transport-related genes are key selection targets driving the *indica*–*japonica* differentiation and breeding improvement of Zhejiang rice.

### 2.5. Genome-Wide Association Studies of Agronomic Traits

**Figure 6:**
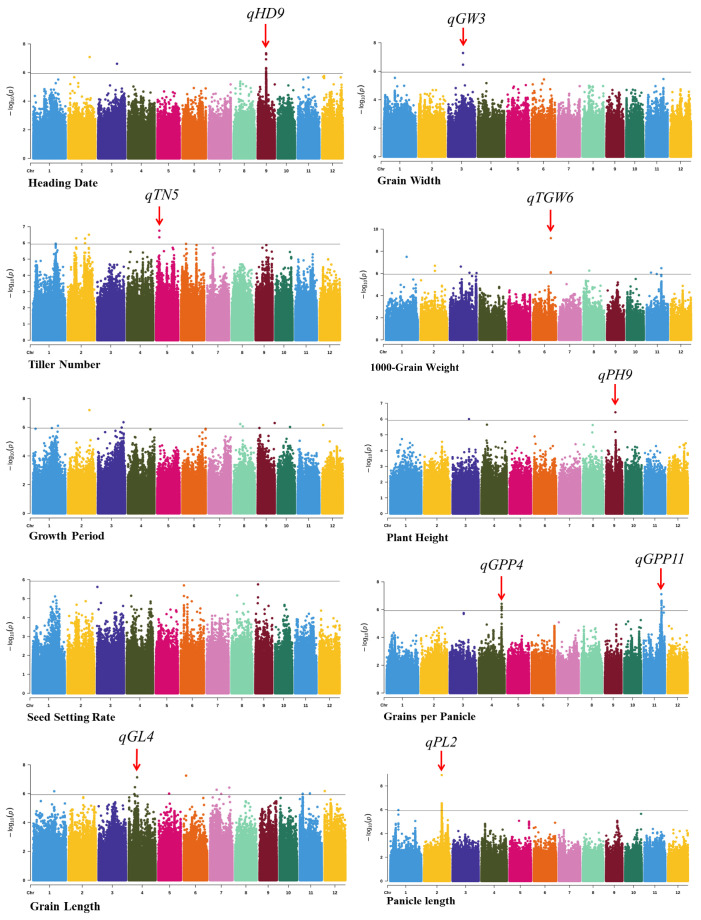
Manhattan plots of GWAS for the agronomic traits. Manhattan plots for ten agronomic traits. The genome-wide significance threshold (–lg*p* = 5).

Through genome-wide association analysis, a total of nine loci significantly associated with agronomic traits were identified among the 109 Zhejiang rice accessions, controlling heading date, effective tiller number, grain length, grain width, 1000-grain weight, plant height, grains per panicle (two loci), and panicle length, respectively (Figure 6 and Figure S3). Among these, the heading date locus (*qHD9*) was mapped to the protein kinase gene *MDPK* (*LOC_Os09g18594*), which is associated with sheath blight resistance [21]. The effective tiller locus (*qTN5*) harbored a candidate gene (*qTN5.10*) whose homologous sequence is involved in the regulation of tillering and plant height. The grain width locus (*qGW3*) contained the magnesium chelatase subunit gene Chl9 (*LOC_Os03g36540*), which affects chloroplast development [22]. The 1000-grain weight locus (*qTGW6*) was associated with genes such as *OsSPX1* (phosphate homeostasis) [23], *MOC1* (tillering and grains per panicle) [24], and *OsAld-Y* (photosynthesis) [25], potentially regulating grain filling through source–sink relationships. The plant height locus (*qPH9*) had the candidate gene *OsSAP17* (*LOC_Os09g21710*), which is involved in stress responses [26]. One grains-per-panicle locus (*qGPP4*) contained the Remorin family gene *GSD1* (affecting seed setting rate and grain width) [27], while the other (*qGPP11*) harbored a receptor-like kinase gene. The panicle length locus (*qPL2*) featured the candidate gene *hbd2*/*LTG1* (*LOC_Os02g40860*) [28], a casein kinase I gene, along with an ammonium transporter and a phenylalanine ammonia-lyase gene, suggesting that nitrogen metabolism and secondary metabolism may participate in panicle development. These results provide important clues for deciphering the genetic basis of yield and adaptive traits in Zhejiang rice germplasm and offer candidate gene resources for molecular breeding.

### 2.6. Gene-Based Haplotype Analysis of Core Candidates

**Figure 7:**
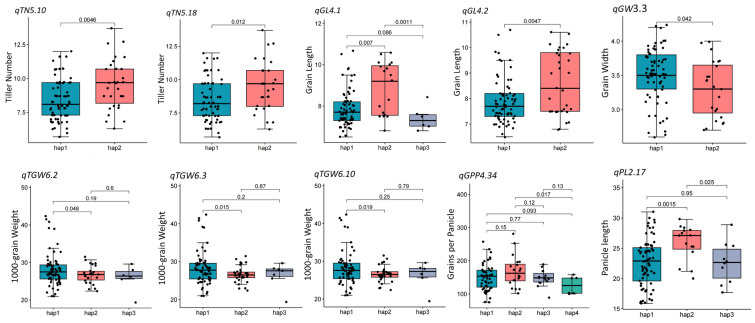
Haplotype analysis of candidate genes associated with agronomic traits.

To dissect the contribution of different allelic variations in candidate genes to agronomic traits, we performed haplotype analyses on candidate genes within each associated interval and identified multiple genes and their elite haplotypes significantly associated with agronomic traits (Figure 7, Supplementary Table S4). The results showed that different haplotypes of the effective tiller number-related genes *qTN5.10* and *qTN5.18* exhibited significant phenotypic differences, suggesting their involvement in tiller regulation. For grain length, haplotypes of *qGL4.1* and *qGL4.2* had significant effects, with specific haplotypes showing marked changes in grain length. Regarding grain width, haplotype analysis revealed that different haplotypes of *qGW3.3* caused highly significant differences in grain width (*p* < 0.01), whereas no significant phenotypic difference was detected among haplotypes of *qGW3.4* (*Chl9*) within the same interval, indicating that *qGW3.3* is the key candidate gene driving phenotypic variation at this locus. For 1000-grain weight, haplotypes of *qTGW6.2* (*OsSPX1*), *qTGW6.3* (*OsWR2*) [29], and *qTGW6.10* (*OsEBF1*) [30] showed significant effects, with the elite haplotypes of the first two significantly increasing 1000-grain weight. Among the grains-per-panicle loci, only the haplotype of *qGPP4.34* (*OsNUC1*, a nucleolar protein gene [31]) significantly affected grains per panicle. For panicle length, haplotypes of *qPL2.17* (*OsCER1*) exhibited significant differences, indicating that *OsCER1* is an important candidate gene for panicle length regulation. These results provide important targets for subsequent gene functional validation and marker-assisted breeding.

## 3. Discussion

Genomic-level characterization clearly partitioned the 109 Zhejiang rice accessions into distinct *indica* and *japonica* lineages, which is highly consistent with the classical subspecific divergence model of Asian cultivated rice. This sharp separation suggests that the *indica* and *japonica* gene pools in Zhejiang have maintained remarkably high genetic isolation with minimal intermediate types, a pattern that likely reflects pronounced ecological zoning within the region that restricts natural hybridization [3,16]. Furthermore, it should be noted that this study was conducted across two ecotypes (*indica* and *japonica*) under the specific environmental conditions—including light intensity, temperature, soil type, and water management—characteristic of the Zhejiang rice-growing region. The observed genomic and phenotypic patterns may therefore reflect the adaptive trajectories shaped by this particular ecological context. Intriguingly, however, our study detected a more complex, hierarchical subpopulation stratification specifically within the *japonica* cluster, offering a fresh perspective on regional evolutionary dynamics. Large-scale genomic resources such as the 3010 Rice Genomes Project have provided invaluable foundations for population genetic analyses and the dissection of complex traits at the global scale [13]. However, these resources are primarily designed to capture global diversity and often lack the resolution to detect region-specific genetic signatures shaped by local breeding histories and ecological conditions. In contrast, our study focuses on a provincial-scale germplasm collection from Zhejiang, which enables the identification of locally enriched allelic variants and selection targets that may have been overlooked in global panels. This regional focus thus complements large-scale efforts by providing fine-scale genomic resources tailored to the ecological and breeding context of a specific rice-growing region.

By executing a genome-wide joint scan of the fixation index (*F*st) and nucleotide diversity (*Pi*) ratios, we detected prominent selective sweep signals at the *qSS1*, *qSS10*, and *qSS12* loci, uncovering the genomic foundation of localized ecological adaptation. Crucially, the *qSS12* selective sweep interval harbors *OsSUT2* [18,19], which encodes a tonoplast-localized sucrose transporter that regulates photo assimilate transport from source leaves to developing grains. The exceptionally strong selection signal captured at this locus demonstrates that optimizing source-to-sink carbon allocation has been a primary target of artificial selection in the history of Zhejiang rice breeding. Mechanistically, the selection at *qSS12* may be linked to the function of *OsSUT2*. This tonoplast-localized sucrose transporter mediates vacuolar sucrose export to the cytosol [32]. Its disruption leads to soluble sugar accumulation in source leaves, causing feedback inhibition of photosynthesis, while simultaneously limiting carbon supply to roots, which inhibits root growth and alters hormone balance. The co-selection of these loci thus likely reflects an adaptive strategy to balance carbon fixation and utilization. Furthermore, the simultaneous clearing of *qSS1* (harboring photosynthesis-related genes) and *qSS10* (harboring the plastidial Clp protease gene *OsCLPP4*, which is involved in photosystem repair) provides robust evidence that enhancing photosynthetic efficiency and maintaining chloroplast homeostasis served as core evolutionary drivers. Collectively, these findings suggest that the simultaneous improvement of source strength and sink capacity—a principle that later became central to the Green Revolution—may have been unintentionally selected through traditional breeding practices in Zhejiang rice germplasm, consistent with the possibility that such selection predates the modern molecular breeding era. Moreover, the co-selection of these loci likely reflects an adaptive strategy to balance carbon fixation and utilization.

Our genome-wide association study (GWAS) mapped 9 significant loci, several of which co-localized with well-characterized genes, validating the robustness of our statistical mapping approach. For instance, the candidate gene for the panicle length locus *qPL2*, *LTG1* (encoding a casein kinase I) [27], has been documented to modulate plant height and grain shape via the brassinosteroid signaling pathway, while the 1000-grain weight locus *qTGW6* was associated with *OsSPX1* (phosphate homeostasis regulation) and *MOC1* (tillering control) [22,23], both of which possess well-established roles in regulating yield traits. Nevertheless, this study also yielded several unexpected insights that challenge or expand current paradigms. Although the grain width locus *qGW3* resided within an interval containing the magnesium chelatase gene *Chl9* [21], gene-based haplotype analysis revealed that the phenotypic variation in grain width was not driven by *Chl9*, but rather by an adjacent FAD-binding protein gene (*qGW3.3*). This exclusionary finding highlights the necessity of coupling high-resolution haplotype dissection with functional validation to avoid false attributions. Furthermore, the effective tiller number locus *qTN5* pointed to a candidate gene (*qTN5.10*) encoding a DUF630/DUF632 domain-containing protein; notably, this domain is plant-specific and remains entirely uncharacterized. Additionally, another candidate gene within the panicle length locus *qPL2*, *OsCER1* (encoding a wax synthase), was previously reported to participate primarily in cuticular wax biosynthesis and male fertility [31]. Our study suggests a potential involvement of *OsCER1* in panicle length regulation, implying that wax metabolism may modulate panicle elongation, possibly through affecting rachis cuticle development or hormone signaling crosstalk. Given that agronomic traits are inherently complex—involving local hormonal balance, epigenetic modifications, and multi-pathway regulation—further investigation is required to determine whether the effect of *OsCER1* on panicle length is direct or mediated through its interaction with other pathways.

Despite these insights, certain limitations of this study warrant consideration. Although the sample size of 109 accessions provided sufficient statistical power to detect major-effect QTLs, its efficacy in capturing rare allelic variations remains limited. Future investigations should expand the population size, integrate multi-year and multi-environment phenotypic data, and perform functional validation of the novel candidate genes—such as the DUF630/DUF632 domain protein gene and the FAD-binding protein gene—via transgenic approaches or *CRISPR*/*Cas9-*mediated gene editing. In conclusion, this study not only establishes a high-resolution genomic variation map of Zhejiang rice germplasm but, more importantly, deciphers the central roles of biological processes such as carbon metabolism, energy homeostasis, and wax biosynthesis in localized adaptation and yield formation from the perspectives of evolutionary selection and agronomic improvement. These findings deliver immediately applicable molecular targets and a solid theoretical framework for precision molecular design breeding in rice.

## 4. Materials and Methods

### 4.1. Plant Materials

A representative panel of 109 rice accessions from Zhejiang Province was utilized in this study (Supplementary Table S5). Field evaluations were conducted over two consecutive growing seasons (2023 and 2024) at the experimental farm of the Zhejiang Academy of Agricultural Sciences. Planting followed a randomized complete block design (RCBD) with three blocks (i.e., three replicates per accession), and standard agronomic management practices were applied uniformly from sowing to harvest. All accessions were grown in the same field under consistent management conditions, including uniform light intensity, irrigation, fertilization, and soil redox potential, to ensure that phenotypic differences among genotypes primarily reflected genetic variation rather than environmental heterogeneity.

### 4.2. Phenotypic Evaluation

Phenotypic data collection targeted ten agronomic parameters. For each block, five representative plants per accession per block were selected. Vegetative-phase records included growth period and heading date (50% panicle emergence per plot). At maturity, in-field measurements encompassed plant height (base to longest panicle tip), panicle length, and effective tiller number. Post-harvest assessments of grains per panicle, seed setting rate, 1000-grain weight, grain length, and grain width were conducted using an automated seed phenotyping system.

### 4.3. Resequencing and Variant Calling

DNA isolation from freshly harvested young leaf tissue employed the conventional CTAB approach. Whole-genome shotgun sequencing on the Illumina HiSeq platform produced an average read depth of ~10× per accession. Following adapter trimming and quality assessment, clean reads were mapped onto the MSU v7.0 *Oryza sativa* ssp. *japonica* cv. Nipponbare assembly via BWA-MEM (v0.7.17-r1188) with parameters -M -R -K 10000000; alignment file manipulation was conducted using Samtools (v1.9) [33].

Single-nucleotide polymorphism detection followed the GATK4 HaplotypeCaller-GVCF paradigm, with cross-sample joint genotyping performed to consolidate individual-level gVCFs into a unified call set [34]. Variant-level quality control utilized GATK4’s variant filter (QD2, QUAL30, FS60, MQ40, MQRankSum-12.5, ReadPosRankSum-8), and subsequent filtering by VCFtools (maf ≥ 0.05, max missing 0.8) yielded a final high-quality biallelic SNP set [35]. No genotype imputation was performed for missing data prior to GWAS analysis.

### 4.4. Population Structure and Genetic Characterization

The population genetic architecture of the 109 Zhejiang accessions was characterized through a three-pronged analytical approach. Dimensionality reduction via PCA was implemented in PLINK (v1.9), with subsequent visualization of eigenvector scores performed using ggplot2 in R. Model-based ancestry inference was conducted with ADMIXTURE (v1.3.0) by iteratively testing ancestral cluster numbers (K) ranging from 1 to 10 [36]. Evolutionary relationships were depicted through a neighbor-joining tree reconstructed in IQ-TREE (v2.2.5) [37]. Genome-wide linkage disequilibrium decay patterns were further quantified using PopLDdecay (–MaxDist 500 –MAF 0.05 –Het 0.88 –Miss 0.999), with the physical distance at which *r*^2^ declined to 50% of its peak value serving as the population-specific LD threshold.

### 4.5. Selective Sweep Analysis of Subspecific Differentiation

We scanned for selective sweeps between indica and japonica using *F*st and *Pi* ratio statistics calculated in 100 kb windows (10 kb step) via VCFtools [34]. Regions concurrently surpassing the 5% *F*st threshold and showing marked *Pi* ratio divergence were retained as candidate sweeps. Genes within these intervals were pulled from the MSU v7.0 annotation [38] and subjected to GO and KEGG enrichment analyses.

### 4.6. GWAS and Haplotype Mining

We deployed the GEMMA package in R to conduct multi-year GWAS for all measured agronomic traits. Association testing employed the mixed linear model (MLM) framework implemented within GEMMA. To correct for population stratification, principal component analysis was performed using PLINK (v1.9) [36], and the first five principal components (PCs) were included as fixed covariates, together with the kinship matrix (K) estimated by GEMMA as a random effect to effectively control false positives arising from population structure and cryptic relatedness. Manhattan and quantile–quantile plots were rendered using qqman in R 3.4.2 to visualize the association landscapes [39,40]. The significance threshold was set at –log10(*p*) = 5.0. This empirical threshold was selected based on its wide adoption in rice GWAS studies, providing a practical balance between detecting genuine associations and controlling false positives [41,42]. Candidate gene intervals were demarcated as ±200 kb flanking each lead SNP, guided by the local LD decay profile (*r*^2^ = 0.2 at ~250 kb) (Figure S4) [43]. For prioritized genes within these windows, haplotype reconstruction was performed using exonic non-synonymous SNPs; phenotypic divergence among haplotype classes was assessed by one-way ANOVA followed by Duncan’s multiple range test.

## 5. Conclusions

This study deciphers the genomic diversity of 109 Zhejiang rice accessions, revealing sharp *indica*–*japonica* divergence and identifying three selective sweep regions—including the sucrose transporter gene *OsSUT2*—that drive subspecific differentiation. Genome-wide association studies mapped nine loci controlling key agronomic traits, and haplotype analyses uncovered elite allelic variations in *OsSPX1*, *Chl9*, and *OsCER1*. These findings provide a valuable genomic resource and molecular targets for precision breeding of rice.

## Figures and Tables

**Table 1:** Descriptive statistics of ten agronomic traits in 109 Zhejiang rice accessions.

Parameter	Growth Period (d)	Heading Date (d)	Plant Height (cm)	Panicle Length (cm)	Effective Tillers (No.)	Grains per Panicle (No.)	Seed Setting Rate (%)	1000-Grain Weight (g)	Grain Length (cm)	Grain Width (cm)
Maximum	176.00	128.00	174.00	31.00	26.00	281.30	96.80	42.40	10.70	4.20
Minimum	115.00	83.00	63.67	15.90	5.70	75.70	60.20	19.40	6.50	2.10
Mean	147.60	103.40	127.30	23.10	9.20	156.80	84.90	28.10	8.20	3.40
SD	13.86	10.53	26.63	3.89	3.18	41.95	8.01	4.17	1.06	0.41
CV (%)	9.39	10.18	20.92	16.84	34.64	26.76	9.43	14.83	12.93	11.89
Shannon’s genetic diversity index (H′)	1.97	1.99	1.97	1.83	2.01	1.98	2.01	2.00	2.01	1.99

**Table 2:** Summary of selective sweep regions (*qSS1*, *qSS10*, *qSS12*) identified by the top 5% *F*st and bottom 5% *Pi* thresholds.

QTL	CHROM	BIN_START	BIN_END	WEIGHTED_*F*st	*Pi*
*qSS1*	1	33,490,001	33,590,000	0.903304	6.6745 × 10^−4^
*qSS10-1*	10	23,180,001	23,280,000	0.911253	6.50803 × 10^−4^
*qSS10-2*	10	23,190,001	23,290,000	0.927236	1.85686 × 10^−4^
*qSS10-3*	10	23,200,001	23,300,000	0.936335	5.91 × 10^−5^
*qSS12-1*	12	27,500,001	27,600,000	0.912014	7.69295 × 10^−4^
*qSS12-2*	12	27,510,001	27,610,000	0.922366	5.54392 × 10^−4^
*qSS12-3*	12	27,520,001	27,620,000	0.922171	2.29968 × 10^−4^
*qSS12-4*	12	27,530,001	27,630,000	0.912612	5.00 × 10^−5^

## Data Availability

The original contributions presented in the study are included in the article/Supplementary Material. Further inquiries can be directed to the corresponding author.

## Supplementary Materials

The following supporting information can be downloaded at: https://www.mdpi.com/article/10.3390/plants15152368/s1, Figure S1: Frequency distribution histograms of ten agronomic traits. Each panel represents one trait. Figure S2: Venn diagram of the intersection between top 5% *F*st windows and bottom 5% *Pi* windows. Figure S3: Quantile–quantile (QQ) plots of genome-wide association studies for ten agronomic traits in 109 Zhejiang rice accessions. Figure S4: Genome-wide linkage disequilibrium (LD) decay in the 109 rice accessions. LD measured as *r*^2^ plotted against physical distance (kb). Supplementary Table S1: Summary of genomic variants and density distribution across chromosomes. Supplementary Table S2: Functional annotation of high-quality variants identified in 109 rice accessions. Supplementary Table S3: Candidate genes located within the selective sweep regions and their functional annotations. Supplementary Table S4: SNP positions (MSU v7.0) in candidate gene intervals. Supplementary Table S5: List of 109 Zhejiang rice accessions used in this study.
