# Supplementary material for: Genomic Insights into the Genetic Diversity, Selection Signals, and Agronomic Traits of Rice (Oryza sativa L.) Germplasm in Zhejiang Province
Source: Plants (Basel). 2026 Jul 31;15(15):2368. doi: 10.3390/plants15152368 (PMC13468211; doi:10.3390/plants15152368)
Supplement: Supplementary file 1 [file plants-15-02368-s001.zip › Supplementary Figure.pdf]

Supplementary materials

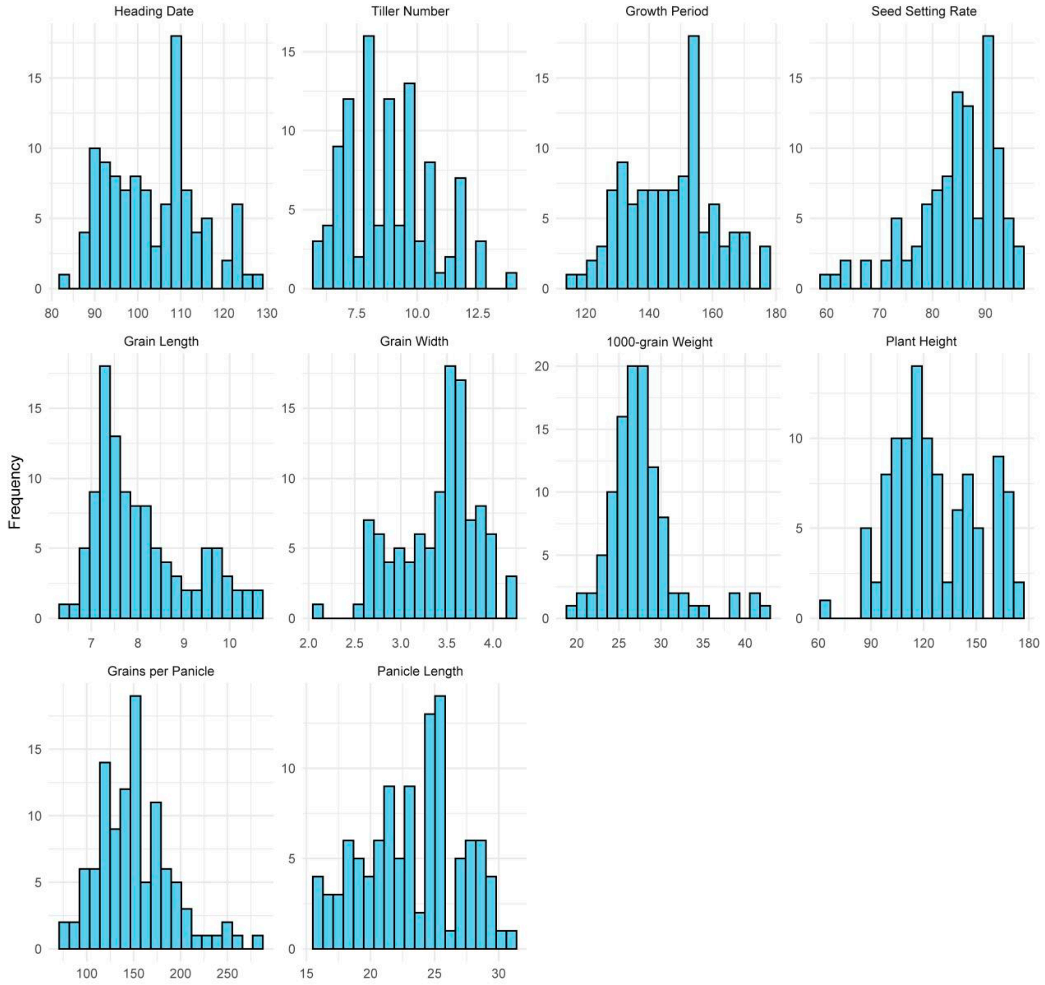

Figure S1. Frequency distribution histograms of ten agronomic traits. Each panel represents one trait.

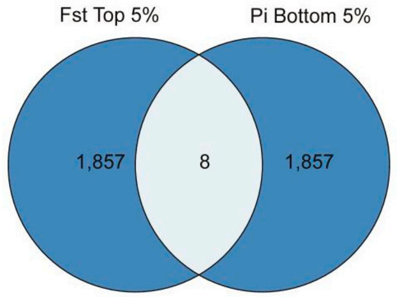

Figure S2. Venn diagram of the intersection between top 5% *F*st windows and bottom 5% *Pi* windows.

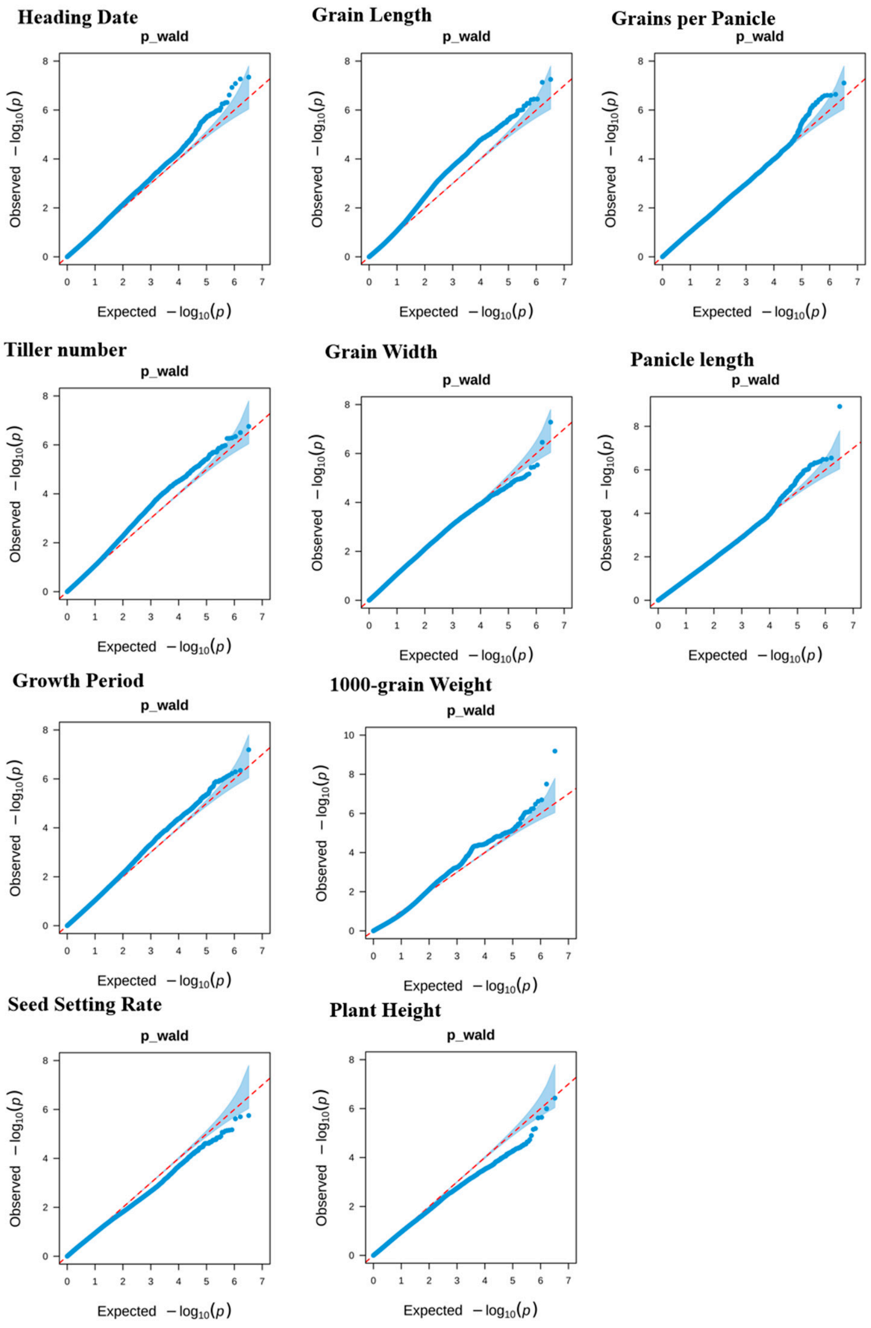

Figure S3. Quantile-quantile (QQ) plots of genome-wide association studies for ten agronomic traits in 109 Zhejiang rice accessions.

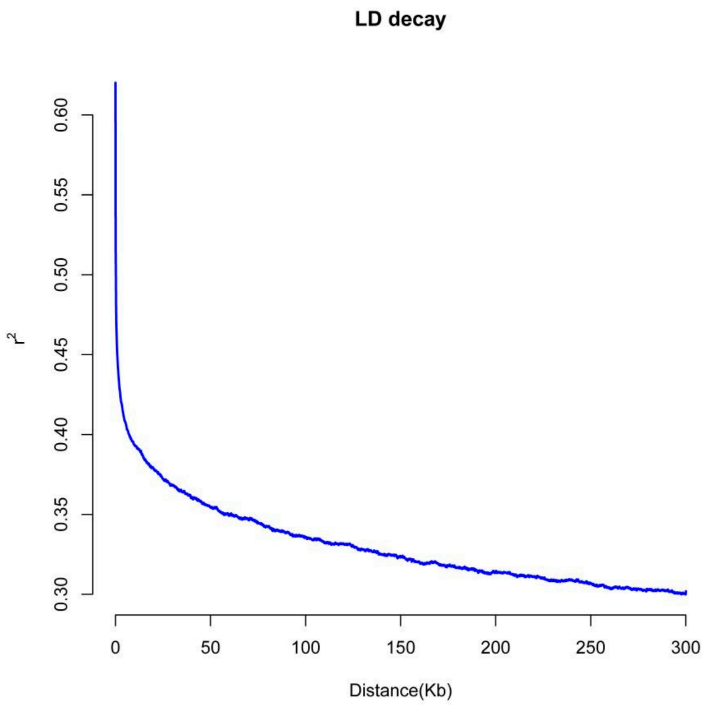

Figure S4. Genome-wide linkage disequilibrium (LD) decay in the 109 rice accessions. LD measured as r^2 plotted against physical distance (kb).
